# The Angiogenic Balance and Its Implications in Cancer and Cardiovascular Diseases: An Overview

**DOI:** 10.3390/medicina58070903

**Published:** 2022-07-06

**Authors:** Cătălina Ionescu, Bogdan Oprea, Georgeta Ciobanu, Milena Georgescu, Ramona Bică, Garofiţa-Olivia Mateescu, Fidan Huseynova, Veronique Barragan-Montero

**Affiliations:** 1 Department of Chemistry, Faculty of Sciences, University of Craiova, 107i Calea București, 200144 Craiova, Romania; geo_ciobanu20@yahoo.com; 2 Histology Department, University of Medicine and Pharmacy, 2-4 Petru Rares, 200349 Craiova, Romania; garo2963@yahoo.com; 3 Clinic for Plastic Surgery and Burns, County Emergency Hospital Craiova, 200642 Craiova, Romania; dr.milenageorgescu@gmail.com; 4 General Hospital—“Victor Babes”, 281 Mihai Bravu St., Sector III, 030303 Bucharest, Romania; bicaramona@gmail.com; 5 LBN, University of Montpellier, 34193 Montpellier, France; fidan.huseynova@etu.umontpellier.fr (F.H.); veronique.montero@umontpellier.fr (V.B.-M.); 6 Institute of Molecular Biology and Biotechnologies, Azerbaïjan National Academy of Sciences (ANAS), AZ1073 Baku, Azerbaijan; 7 Department of Histology, Cytology and Embryology, Azerbaijan Medical University, AZ1078 Baku, Azerbaijan

**Keywords:** angiogenesis, activators, inhibitors, cancer, cardiovascular diseases

## Abstract

Angiogenesis is the process of developing new blood vessels from pre-existing ones. This review summarizes the main features of physiological and pathological angiogenesis and those of angiogenesis activation and inhibition. In healthy adults, angiogenesis is absent apart from its involvement in female reproductive functions and tissue regeneration. Angiogenesis is a complex process regulated by the action of specific activators and inhibitors. In certain diseases, modulating the angiogenic balance can be a therapeutic route, either by inhibiting angiogenesis (for example in the case of tumor angiogenesis), or by trying to activate the process of new blood vessels formation, which is the goal in case of cardiac or peripheral ischemia.

## 1. Introduction

According to the American Heart Association, in 2013, one out of three persons died because of cardiovascular diseases in the United States, while stroke and heart attacks were considered to be the primordial causes of worldwide death [1].

Cancer is another disease affecting many lives, being one of the major and most frequently observed death causes in the European Union, based on statistical reports. What could bring together these diseases that have such different causes and specific molecular mechanisms?

One of the principles that could somehow “bridge” them is angiogenesis regulation. By inhibiting tumor angiogenesis, anticancer treatments become more effective whereas, on the contrary, by activating angiogenesis, the formation of new blood vessels would lead to a better vascularization of ischemic heart regions.

Due to its implications of the utmost importance, a great deal of research has been devoted to angiogenesis and its underlying mechanisms. Physiological angiogenesis occurs as a result of a fine-tuned balance of endogenous regulators, having, mostly, a protein structure. The actions of activators, such as fibroblast growth factor (FGF), vascular endothelial growth factor (VEGF), “platelet-derived endothelial cell growth factor” (PD-ECGF), angiopoietin, and angiogenin [2,3,4,5,6], are balanced by processes determined by inhibitors, such as endostatin and angiostatin. The vascular endothelial growth factor (VEGF) action is closely related to the stimulation of angiogenesis [5,6]. The VEGF proteins family consists of six members [5,7,8], with VEGF-A being the most studied, included in certain anti-cancer therapies [7,9]. Exogenous compounds such as polypeptides [10] or small synthetic molecules, antibiotics, saccharides and steroids have been shown to induce angiogenesis regulation [11,12].

Disequilibrium of the balance of angiogenesis regulators leads to insufficient angiogenesis, linked to ischemia, ulcers, infertility or hair loss. Excessive angiogenesis has been identified as being related to tumor growth, metastasis appearance or to a series of other diseases, among which is age-related macular degeneration, associated with elevated VEGF levels. Therefore, the therapeutic approach of anti-angiogenic therapy involves inhibitors that are able to recognize and block either VEGF itself or its receptors. On the other hand, therapeutic angiogenesis may be induced through protein, gene or cell therapy. Careful clinical studies are being conducted, as systemic effects must be avoided.

Based on the above, our mini-review is organized in sections, as follows: formation and remodeling of blood vessels; the angiogenic balance—synthetic and endogenous regulators; physiological and pathological angiogenesis; angiogenesis inhibition and current implications in cancer treatment; angiogenesis activation and current implications in cardiovascular diseases.

## 2. Formation and Remodeling of Blood Vessels

Blood vessels that supply the body with oxygen and nutrients, are formed through three different mechanisms (vasculo-genesis, angiogenesis, arterio-genesis) united under the name of neovascularization.

Vasculo-genesis. Blood vessels are formed in the early stages of embryo development through vasculo-genesis when the embryonic mesenchymal cells differentiate into endothelial cells and a “primary capillary plexus” is formed. At present, it is known that, besides its role in embryonic development, vasculo-genesis is also induced after birth. In this case, bone marrow-derived endothelial progenitor cells mediate the cases of physiological and pathological neovascularization, for example, in the cases of wound healing or cardiac ischemia [13,14,15].

Angiogenesis is the main process in post-natal neovascularization and represents the process of new vessel formation from pre-existing ones [16], through two mechanisms: intussusception [17,18] and sprouting [19]. Intussusception represents the “splitting” of an existing vessel into two new vessels, with interior reconstruction of novel blood vessel walls. Leading to a rise in the number of vessels without augmentation of the number of endothelial cells, intussusception angiogenesis is involved in the remodeling of existing vessels, for example, in capillary formation starting from the primary plexus in embryo development, but it can also be seen in post-natal development. Sprouting angiogenesis is a more complicated process. It is regulated by different angiogenic factors and involves several steps: degradation of the basement membrane of the vessel under enzymatic conditions, endothelial cells activation, proliferation and migration, formation of a new lumen and pericyte stabilization [19,20,21,22]. There is much evidence in different studies that a tumor’s vascularization is associated with the metastatic risk and negatively influences the survival rate. The micro-density of a tumor’s vascularization is especially used for the follow-up of anti-angiogenetic therapy [23].

Arterio-genesis represents the formation of new blood vessels from co-existing, co-lateral vessels, with the participation of smooth muscle cells, as an adaptive process to an arterial occlusion [24,25], being rather a re-modeling process. In the proximity of an arterial stenosis, the substitution network is architectured through both arterio-genesis (in near regions, unleashed by mechanical constraints and cytokines) and angiogenesis (in distal sites, where hypoxia would generate new vessel sproutings) [26,27].

## 3. The Angiogenic Balance: Synthetic and Endogenous Regulators

### 3.1. Synthetic Modulators of Angiogenesis

Most of the endogenous molecules playing a role in angiogenesis modulation have protein structure, but their usage in therapy is delicate, because of the high cost of their production in large quantities and because of the difficulty in penetrating tissues.

For this reason, more attention has been shown to the preparation and investigation of certain compounds impacting the angiogenesis, such as the polypeptides with therapeutic effect [10]. In the same terms, relating to angiogenesis modeling, some antibiotics, polysaccharides, steroids, and other synthetic small-molecular compounds have been reported [11,12].

As our research area is represented by carbohydrates, we review in detail this type of compounds used as angiogenesis modulators. Many carbohydrate-binding proteins are involved in angiogenesis; therefore, carbohydrates and their analogues may be important factors for angiogenesis regulation [28].

One example is represented by galectin-3 and MCP (modified citrus pectin). Galectin-3 is a β-galactoside-binding lectin, which mediates endothelial cell morphogenesis in vitro and angiogenesis in vivo [29].

Nangia-Makker et al. proved that it is able to tightly bind to galectin-3, via recognition of its carbohydrate recognition domain, and to inhibit angiogenesis and tumor growth [30]. Johnston at al. synthesized and studied heparan sulfate mimetics, represented by a series of poly-sulfated penta- and tetra-saccharide glycosides containing alpha(1→3)/alpha(1→2)-linked mannose residues. They found that the investigated mimetics bound tightly to angiogenic growth factors and exhibited potent activity indicative of angiogenesis; they strongly inhibited heparanase activity and also showed good antitumor activity [31].

Our research group has proved that synthetic mannose-6-phosphate analogues can act as angiogenesis activators or inhibitors, depending on the structure of the chemical group functionalizing the C6 position of mannose [32,33]. In another study, we prepared gold nanoparticles decorated with various mannose derivatives functionalized in the C6 position and they proved to be effective over angiogenesis [34]. The cation-independent mannose-6-phosphate receptor was previously indicated as inducing angiogenesis through several possible mechanisms [35], but this was the first time that mono-carbohydrates have directly been indicated as an agent possessing angiogenic activities.

Since then, it has been proven that 1,2,3,4,6-penta-O-galloyl-β-d-glucopyranose (PGG) has antiangiogenic activity in vitro and in vivo. Derivatives of PGG with different sugar cores and phenolic substituents have been tested and they are also angiogenesis inhibitors [36,37,38].

### 3.2. Endogenous Regulators of Angiogenesis

Besides mechanical (shear stress and blood flow augmentation) and chemical (hypoxia and nitric oxide increase) influences, angiogenesis is regulated by molecular influences, among which the most important are the angiogenic growth factors: fibroblast growth factor (FGF), vascular endothelial growth factor (VEGF), “platelet-derived endothelial cell growth factor” (PD-ECGF), angiopoietin; angiogenin, etc. [2,3,4]. The vascular endothelial growth factor (VEGF) action is closely related to the stimulation of angiogenesis [5,6]. Angiogenesis is, on the other hand, inhibited by anti-angiogenic factors, such as angiostatin, endostatin, thrombospondin-1 (TSP-1), heparinases, etc. [39]. When the balance between angiogenesis activators and inhibitors loses equilibrium, abnormal (either insufficient or excessive) angiogenesis occurs and various diseases appear or degenerate [40].

The main endogenous angiogenesis activators are summarized in Table 1. The most commonly studied endogenous angiogenesis activators are the FGF and VEGF families. bFGF is the first angiogenic factor that has been purified, in 1975 [41], and FGFs are the first angiogenic factors that were sequenced, in 1985 [42]. VEGF has been identified in 1983 as a vascular permeability factor [43], and only later, in 1989, has it been shown to possess angiogenic action [44]. Table 2 summarizes the main endogenous angiogenesis inhibitors. Thrombospondin-1 is the first protein observed to possess naturally occurring antiangiogenic properties [45,46]. Since then, the number of angiogenesis regulators have grown and, besides endogenous regulators, synthetic molecules with effect on angiogenesis have been obtained and tested, some of them already on the market and available for treatment.

There are studies that prove the binding of growth factors to the cell surface, serving as target, receptors, or even as a storage mechanism. This seems to be valid for the inductor FGF which induces the activation of VEGF.

The class of VEGF proteins consists of several derivatives such as VEGF-A, VEGF-B, VEGF-C, VEGF-D, VEGF-E (encoded-virus) and VEGF-F (derived from snake venom) and placental growth factor (PlGF) [5,7,8]. Vascular permeability and inflammation, angiogenesis and apoptosis, lymphangio-genesis and fibrogenesis can be adjusted by the VEGF family [8]. From all the VEGF class components, VEGF-A is the most individualized, representing a substantial angiogenesis promotor and consequently designed as an objective for the study of certain anti-cancer therapies [7,9]. According to the chain length, different VEGF-A isoforms have resulted after the splicing of alternative VEGF mRNA [8], and have further been referred to as VEGF_XXX_, where “XXX” indicates the number of amino acids from the final protein chain [7]. The most well-known subtypes are VEGF_111_, VEGF_121_, VEGF_145_, VEGF_165_, VEGF_189_ and VEGF_206_ [5]. 

Human VEGF-A contains eight exons separated by seven introns, all their subtypes presenting similar regions, namely exons 1–5 and 8 [8]. The longer VEGF isoforms containing both exons 6a and 7, such as VEGF_145_, VEGF_189_ and VEGF_206_ have high affinity for heparin sulphate glycoproteins [5,7,47,48]. VEGF_162_ is a VEGF isoform, whose protein sequence has exons 1–5, 6a, 6b and 8. proliferating the angiogenesis in vivo, while VEGF_165_ is the most potent endothelial cells proliferation agent. The VEGF shorter isoforms, such as VEGF_111_ and VEGF_121_, do not have exons 6 and 7, are highly diffusible and. therefore, cannot connect to the extracellular matrix [5].

The main endogenous activators and inhibitors of angiogenesis are summarized in Table 1 and Table 2.

Certain studies have demonstrated that the thickness of the capillary’s basal lamina may lead to insufficient oxygen diffusion, limiting the elimination of some metabolites, eventually leading to the increase of different diseases’ severity [80,81]. Minchenko et al. [82] suggested that hypoxia is both inductor and stimulator of VEGF expression in vivo, along with the increase of glycemia [83].

VEGF also increases the microvascular permeability that precedes and accompanies angiogenesis, playing a central role in its process, acting as an anti-apoptotic factor for endothelial cells in newly formed blood vessels.

Many studies have highlighted the histological structural changes in the blood vessels of periodontopathic diabetic patients, but less data were reported on the number of abnormally evolution vessels (MVD) and/or their tissue distribution process, which can lead to tumor growth and the development of metastases (neo-angiogenesis).

Thus, in addition to the structural changes visible with light microscopy for the gingival blood capillaries, the number of blood vessels from the gingiva of the periodontal patients with diabetes mellitus (DM) can be displayed by quantitative immunohistochemical method and vascular markers.

**Figure 1:**
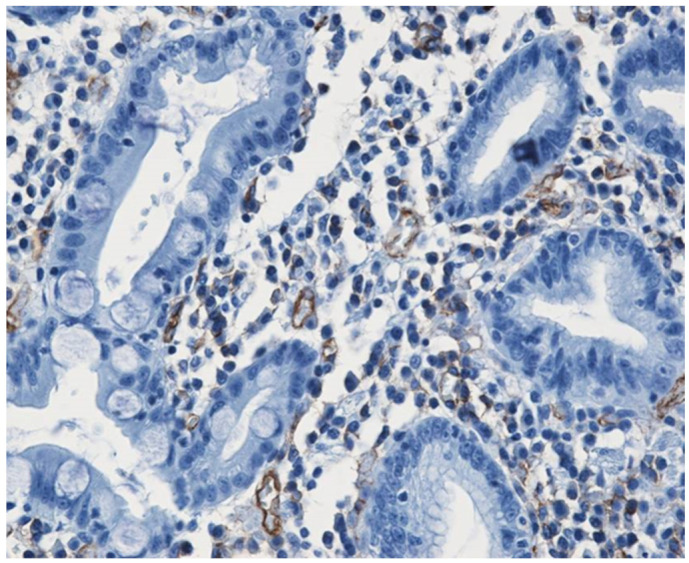
Small caliper blood vessels immunostained for CD34 antibody (DAB ×200).

We believe that the best immunohistochemical marker to highlight the newly formed blood vessels is the CD31+CD34 antibody cocktail which may show the entire micro-vascular network, as shown in Figure 1.

## 4. Physiological and Pathological Angiogenesis

**Figure 2:**
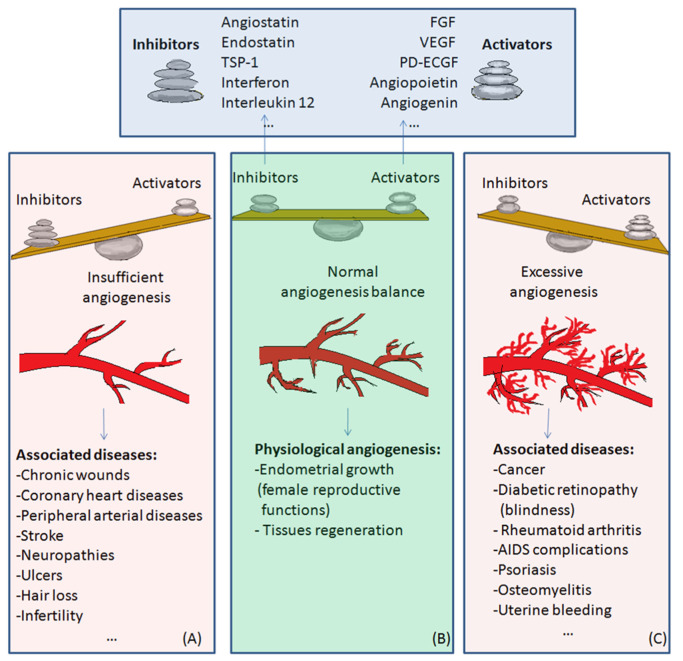
The balance of activators and inhibitors in: (**A**)—insufficient angiogenesis; (**B**)—normal angiogenesis; (**C**)—excessive angiogenesis. Abbreviations: FGF: fibroblast growth factor, VEGF: vascular endothelial growth factor, PD-ECGF: platelet-derived endothelial cell growth factor, TSP-1: thrombospondin-1.

The balance of activators and inhibitors is shown in Figure 2. Besides its role in embryonic development, angiogenesis appears as a normal process in adults, in female reproductive functions [84,85,86,87,88] and in tissue regeneration (ex. wounds healing) [89] (Figure 2B). Under certain conditions, it can appear as a disequilibrium in the synthesis of the endogenous factors that control angiogenesis, and an abnormal angiogenesis can occur. It can be either insufficient, leading to the impossibility of the body healing wounds or participating in normal organ regeneration, or it can lead to the ischemia of a part of the body, such as myocardial, peripheral of intestinal ischemia. Other complications include ulcers, infertility and hair loss (Figure 2A).

As mentioned above, excessive angiogenesis can favor tumor growth and metastasis appearance or it can induce the evolution of diseases such as rheumatoid arthritis, psoriasis, etc. (Figure 2C) [3,16].

Excessive angiogenesis is also linked to a series of eye diseases, which can lead to blindness. It is the cause of visual loss in the case of age-related macular degeneration. Patients who suffer from proliferative diabetic retinopathy are known to have higher VEGF levels when compared to healthy persons [90]. Increased VEGF levels cause uncontrolled angiogenesis in these patients.

Normal angiogenesis is the balanced action of angiogenesis activators and inhibitors. In the case of preponderant action of angiogenesis inhibitors, insufficient angiogenesis appears, leading to diseases such as chronic wounds, cardiovascular diseases, neuropathies, ulcers, hair loss, and infertility (Figure 2A). Physiological angiogenesis appears as a normal process in female reproductive functions and in tissue regeneration (Figure 2B). On the contrary, if the action of angiogenesis activators prevails, complications in diseases such as cancer, diabetic retinopathy, rheumatoid arthritis, AIDS, psoriasis, osteomyelitis or uterine bleeding may appear (Figure 2C).

## 5. Angiogenesis Inhibition (Anti-Angiogenic Therapy) and Current Implications in Cancer Treatment

The study of angiogenesis inhibitors gained much attention after the discovery by Judah Folkman showing that tumors do not grow bigger than 2–3 mm^3^ because of lack of nutrients, without supply of new blood vessels [91]. Indeed, under oxygen deprivation, tumors produce specific pro-angiogenic factors, which lead to the development of a new bold vasculature feeding the tumor and producing its growth, a phenomenon known as tumor angiogenesis [91,92].

Inhibiting the development of new blood vessels in tumors seemed to be an interesting path in fighting cancer. The anti-angiogenic therapy seemed a very promising target, but clinical medicine showed that, after years of practice, “the miracle drug” still did not show up. This might be due to a series of factors linked to the fact that tumors have unique properties that distinguish them from all other tissues.

Tumor vasculature is heterogeneous while tumor blood vessels are larger in size and more permeable than those of normal tissues [93]. The blood pressure is also different inside tumors: it is higher at the periphery, where arteries and arterioles are preponderant, and smaller in the core, where most blood vessels are veins and venules. Therefore, the access of both nutrients and medication through the blood stream is restrained to the margins of the tumor, while the interior, fed through diffusion, becomes necrotic.

A direct consequence of this heterogeneous structure inside tumors is that the liquids tend to accumulate in the interstitium of tumors, creating an increased interstitial fluid pressure (IFP) in the center of the tumor compared to the periphery [94,95]. This is why the liquid flow is oriented from the center to the margins of the tumor and the access of drugs to the tumor core is difficult [96,97]. This affects nanoparticles to a less extent than small drugs. Nanoparticles’ access to the tumor site is possible because blood vessels in tumors are permeable, a phenomenon known as the enhanced permeability and retention (EPR) effect, according to which macromolecules and nanoparticles tend to accumulate more in a tumoral tissue than in a healthy one [98]. The EPR effect represents the basis of the whole generation of nanoparticles intending to reach tumor sites through passive targeting.

All of these factors have made anti-angiogenic therapy an extremely complicated procedure. Two classes of angiogenesis inhibitors may be distinguished: direct inhibitors, that target endothelial cells (ECs), and indirect inhibitors, that target cancer cells or tumor-related stromal cells [99]. Indirect inhibitors act by disrupting the proangiogenic contact between tumor cells and ECs. Even though several mechanisms are known, most of these inhibitors act as anti-VEGF therapies, by blocking either VEGF itself or VEGF receptors (VEGFRs) [100]. These molecules are able to recognize and inhibit VEGF or VEGFRs. In Table 3 are presented the approved angiogenesis inhibitors used to treat cancer in humans, according to the National Cancer Institute [101].

Treatments with anti-VEGF drugs are known to have side-effects (hypertension, proteinuria, thromboembolisms, bleeding, etc.) [117,118] and treatment discontinuity causes vascular reformation in tumors, so new anti-angiogenesis targets are being investigated [119]. In patients treated with Avastin (the trade name for bevacizumab, a monoclonal antibody that acts as an anti-VEGF agent), temporary remission has been observed, but the overall survival time has not been effectively increased [120]. These patients developed resistance to treatment [121] and the investigation of the molecular mechanisms of resistance to anti-angiogenesis treatment has recently attracted great attention [122,123]. Anti-angiogenesis therapy showed better results combined with other treatments, the angiogenesis inhibitors acting in this case as agents that “normalize” the structure and functions of tumor vasculature, facilitating medication (usually chemotherapy) access inside tumors [124,125]. Avastin has been approved by the FDA as treatment for metastatic colorectal cancer combined with chemotherapy [126] and is also used in the treatment of other types of cancer (lung, ovarian, cervix, kidney, brain).

Besides the acquired resistance to chemotherapy and to anti-angiogenic treatments of cancer cells, another intriguing aspect regarding the difficulty of winning the fight against tumors is their incredible ways of finding previously unthinkable resources for opposing the healthy body through surprising “conquering strategies”. Indeed, when tumors exceed a size of several mm3, along with the formation of a new blood vasculature that brings oxygen and nutrients to cancer cells, an opposite phenomenon also takes place: tumor cells fight in order to escape the hypoxic area, and, through mechanisms involving Hepatocyte Growth Factor (HGF), a protein that links to the Met tyrosine kinase receptor, they leave the tumor and fix to distal sites, leading to metastasis [127]. Thus, new directions in cancer therapy tend to combine the administration of anti-angiogenic drugs with the anti-Met treatment [128,129]. The right amount and combination of medication and its well-scheduled administration could hopefully alleviate hypoxia and its non-desired side-effects [124].

The dual inhibition strategy using brivanib (dual FGF/VEGF inhibitor) increased overall survival (OS), and dovitinib (VEGFR, FGFR and PDGR inhibitor) retarded growth of tumors in mice cancer types. On the other hand, VEGF/ANG2 restricting/blocking leads to revascularization suppression and, consequently, the tumor evolution can be stopped, thus increasing OS [6,130].

Although some preclinical prototypes have been shown to be effective in addressing tumor angiogenesis, the complexity of tumor vascularization processes has led to less convenient clinical outcomes [6]. In order to prevent the recurrence of tumors, their surgical resection followed by chemotherapy and/or radiotherapy represents the standard procedure constituting the pathway to an eventual cure [6,131].

## 6. Angiogenesis Activation (Therapeutic Angiogenesis) and Current Implications in Cardiovascular Diseases

Cardiovascular diseases can be divided into cerebrovascular disease, coronary artery disease, peripheral arterial disease and aortic (thoracic or abdominal) atherosclerosis [132]. Usually, the problem is that of an occlusion of a vessel, followed by the ischemization of the surrounding tissue. The required medical intervention aims to restore the normal level of oxygen and nutrients in the affected area, either pharmacologically, or mechanically using surgical interventions (through vascular bypass and angioplasty). Some patients cannot be submitted to these treatments. Others do not respond to treatment as expected and cannot regain a high percentage of revascularization. In all of these cases, methods that could restore natural processes of blood vessel formation are highly welcomed.

Pro-angiogenic therapy would be, in this context, a very interesting opportunity [133]. However, excessive angiogenesis has to be avoided, as it can lead to systemic side-effects, which include the acceleration of diseases such as proliferative retinopathy or atherosclerosis [134].

Therapeutic angiogenesis can be achieved through three routes, as shown below.

- Protein therapy: consists of the repeated administration of angiogenic factors in order to promote angiogenesis. This strategy has the advantage that angiogenic protein production and purification is known, and proteins may now be stored after lyophilization and reconditioned in a buffer upon need. Most of the angiogenic proteins are now commercialized and available for research. Besides VEGF and FGF (the most extensively studied proteins in therapeutic angiogenesis), other factors such as the PDGF family and Angiopoietin-1 have also been investigated. Systemic delivery of proteins has as a drawback the low concentration of the angiogenic factor in the desired tissue, due to both low targeting and rapid protein clearance by the mononuclear phagocyte system. Because of the rapid clearance of proteins in blood, their local delivery, either directly, or using adequate biomaterials with slow-delivery of active principles, is better suited in this case. Local administration (intracoronary, intramyocardial or intracerebral) is possible, but involves the usage of special devices or invasive surgery. Biomaterials of natural or synthetic origin such as hydrogels (e.g., alginate hydrogel and peptide nanofibers); micro- and nano-particles (e.g., poly (lactic acid-co-glycolic acid) microspheres and liposomes); porous scaffolds (e.g., poly (ε-caprolactone) scaffolds); coacervate (e.g., (polycation–heparin) coacervates), have been studied as delivery vehicles of angiogenic proteins [135].
- Gene therapy consists of administrating genes whose expression would lead to proteins that will induce angiogenesis activation [136]. Gene therapy has as an advantage the fact that the protein continues to be secreted a long time after drug administration, as well as the fact that genes might be targeted to specific tissues [137]. Employed vector systems are plasmids and viral vectors. Adeno-associated viruses have been investigated as promising new vectors for gene therapy. The obstacles that have to be overcome are related to the low concentration of gene product at the target site as well as the activation of inflammatory and immune responses. Additional aspects, such as the incomplete understanding of angiogenesis mechanisms at molecular level and the difference between animal models and humans, represent the main obstacles encountered when angiogenesis gene therapy has been applied to humans. Although not yet implemented in clinical practice, data gathered in more than 20 years of preclinical and clinical studies has brought great insights and advancement in this field [138,139]. Several clinical trials investigating the effect of different gene therapies on cardiac regeneration are currently ongoing [140].
- Cellular therapy [141,142] induces angiogenesis using cells known to produce angiogenic factors, such as monocytes and endothelial progenitor cells. Recent research indicates that stem cells-derived extracellular vesicles promote angiogenesis in cellular experiments and animal models. Extracellular vesicles transport informational molecules, including proteins, mRNA, microRNAs, DNA fragments, and lipids [143,144,145,146].

The ability of VEGF and FGF factors to induce therapeutic angiogenesis has been widely studied in preclinical and clinical trials [147]. Through vascular permeability increase and incorporation of endothelial cells, VEGF promotes angiogenesis [8]. The stimulation of endothelial cell layer growth covering the inner lumen of blood vessels, having an effect on the blood supply regularization according to local requirements, leads to the damaged vascular network reconstruction [148,149,150]. Thus, a good strategy for therapeutic angiogenesis can be designed in this direction [148,149,150]. It is considered that the “truthful” endothelial precursor is represented by endothelial colony forming cells (ECFCs) [148,151], which play an important role in conserving endothelial homeostasis and ameliorating ischemia impact on human health by regulating the local blood flow [148].

Studies indicate that cells with vasculo-reparative properties may represent an interesting target for potential therapies in vascular injury and impaired regenerative responses [152].

As shown in a recent review [148], clinical trials have accurately shown that cell therapy based on transplantation of myeloid EPCs fails to provide a significant improvement in capillary density and local blood flow in the case of patients with Cardio Vascular Diseases (CVD) [148].

Cardiac myocytes, known as cardiomyocytes (CM), are the muscle cells which compose the heart muscle and constitute a VGEF-A source that carries out certain functions of the heart [48]. In the case of rat CM, the mechanical stress controls VEGF-A expression and stretching contributes to secretion improvement, [48], involving hypoxia-inducible factor 1-α [48,153]. The absence of transcription factor, GATA-4 protein, that directly links VEGF-A. highlighted a low capillary density of mice hearts, while GATA4 overexpression led to vascularization regulation and cardiac function improvement after myocardial infarction [48].

Clinical trials show that therapeutic angiogenesis is generally safe and does not produce side-effects [154], but accidents may occur [155]. Initial results looked very promising, but most of the clinical trials were considered to be inconclusive. One drawback of reported clinical trials has been the lack of large enough control groups [156], while other clinical trials did not provide good enough positive results, as previously reviewed [157,158]. In future clinical trials, larger randomized groups are expected to be enrolled in order to obtain significant results and long-term studies still need to be finalized in order to have solid conclusions [159].

## 7. Conclusions

Normal angiogenesis is the exact balance of angiogenesis activators and inhibitors and its disequilibrium leads to life-threatening diseases or to diseases seriously affecting patients’ life-quality. In the beginning, a huge amount of attention was dedicated to the inhibition of angiogenesis, mainly in order to inhibit tumor angiogenesis. More recently, therapeutic angiogenesis has attracted great attention, due to its applications mainly in the field of ischemic tissue recovery.

The results of clinical trials prove that anti-angiogenic therapy alone will not be able to effectively treat cancer. Further insights into the molecular mechanisms of cancer resistance to anti-angiogenic therapy will provide important knowledge in this field. Future treatments for cancer can be successful only by combining different types of medication, through well scheduled therapeutic schemes that should try to overcome current limitations.

Therapeutic angiogenesis is under study, the most promising strategy being protein therapy or a combination of treatments involving protein therapy. The implementation of new biomaterials with specific local-delivery properties will probably bring important advancements in this field.

## Figures and Tables

**Table 1:** Endogenous angiogenesis activators.

Activator	Description/Structure	Receptor(s)/Cellular Targets	Mechanism of Action	References
FGF family	- group of over 20 members; - the most studied members are: aFGF (FGF1) and bFGF (FGF2); - small polypeptides of 155–268 amino acids.	- group of receptors, four of them (FGFR1 to FGFR4) being the most studied; - belong to the tyrosine kinase superfamily and are known to dimerize; - FGFRs are composed of an extracellular domain that binds the ligand, having three immunoglobulin-like domains (D1-D3), a single transmembrane helix domain and an intracellular tyrosine-kinase domain. - FGFs binding to FGFRs is influenced by FGFs interaction with HSPG associated to cell surface.	- FGFs are strong mitogens of different cell types	[4,49,50,51,52,53]
VEGF family	- group of six members: VEGF-A (commonly termed as VEGF); VEGF-B; VEGF-C; VEGF-D; VEGF-E; PlGF. - VEGF-A isoforms: - VEGF_121_, VEGF_145_, VEGF_162_, VEGF_165_, VEGF_165B_, VEGF_183_, VEGF_189_ and VEGF_206_.	- group of 3 receptors (VEGFR1 to VEGFR3) - like FGFRs, they belong to the tyrosine kinase superfamily and are known to dimerize; - VEGFRs are composed of an extracellular domain having seven immunoglobulin-like domains, a transmembrane domain and an intracellular tyrosine-kinase domain.	- mitogenic action restricted to endothelial cells; - VEGF-A is a vasodilator and was initially referred to as VPF because of its capacity of increasing microvascular permeability.	[3,4,54,55,56]
Ang 1	- belongs to the angiopoietins’ group, along to Ang2, plus other several angiopoietin-related factors; - Ang 2 is an antagonist of Ang1; - Ang 1 is a protein formed of 498 amino-acid residues (57 kDa);	- Ang1 binds to TIE2 receptor; - Ang1 multimerizes (the tetramer level of four or higher must be archived) prior to receptor binding; - TIE2 heterodimerizes with TIE1 in order to exhibit its biological activity	- mediates endothelial cells’ migration, adhesion and survival and plays an essential role in vessel maturation	[4,57,58,59,60]
Ephrins	- group of 8 proteins linked to cell membrane; - ephrins are divided into two subgroups: - group A (5 ephrin ligands: ephrin A1–A5) and group B (3 ephrin ligands: ephrin B1–B3)	- group of receptors (Ephs) divided into two subclasses, depending mainly on the type of ligand: EphsA and EphsB; - Ephs are the largest subfamily of RTKs and dimerize upon ligand binding.	- Eph recognition by Eph receptors mediates cell adhesion to extracellular matrix, juxta-crine cell–cell contacts, and cell migration.	[4,61,62,63]
MMPs	- zinc-dependent endopeptidases (protein structure) also known as matrixins; - MMPs can be divided into secreted MMPs (gelatinases, strome-lysins, collagenases, other secreted MMPs) and membrane-bound MMPs.	- Secreted Mms interact with membrane–bound MMps, activated, at their turn, intracellularly. - PAR1 (a G protein-coupled receptor involved in various types of cancer) is known as receptor of MMP-1.	- MMPs are involved in tissue remodeling, by degradation of the extracellular matrix.	[4,64,65,66]

Abbreviations: FGF—fibroblast growth factor; aFGF—acidic FGF; bFGF—basic FGF; FGFR—FGF receptor; HSPG—heparan sulfate proteoglycans; VEGF—vascular endothelial growth factor; VEGFR—vascular endothelial growth factor receptor; PlGF—placental growth factor; Ang 1—Angiopoietin 1; Ang 2—Angiopoietin 2; TIE2—tyrosine kinase with immunoglobulin and epidermal growth factor homology domain 2; Ephs—ephrin receptors; RTKs—receptor protein tyrosine-kinase; MMPs—Matrix metalloproteinases; PAR—Protease-activated receptor.

**Table 2:** Endogenous Angiogenesis inhibitors.

Inhibitor	Description/Structure	Receptor(s)/Cellular Targets	Mechanism of Action	References
Endostatin	- Fragment of collagen XVIII; - It can exist as a monomer or as a trimer, in soluble or insoluble form;	- Endostatin binds with low affinity to all surface heparan sulfate proteoglycans involved in growth factor signaling and it also binds to heparin;	- it inhibits certain MMPs; - it reduces the invasion of tumor cells and of ECs, with no effect on proliferation.	[39,67,68,69]
Tumstatin	- Fragment of type IV collagen (28kDa);	- Tumstatin binds to integrins (ex. αvβ3)	- it inhibits the proliferation of ECs and promotes apoptosis with no effect on migration of ECs.	[39,70]
Angiostatin	- Plasminogen fragment (it is known that plasminogen itself doesn’t possess antiangiogenic properties);	- Angiostatin and plasminogen bind to α_v_β_3_ integrin; - it binds to ATP synthase at the surface of endothelial cells; - Angiomotin is a protein known to bind and to internalize angiostatin.	- Angiostatin inhibits ECs’ proliferation and migration.	[39,71,72,73]
TSPs	- TSPs are divided into two subgroups: subgroup A (including TSP-1, the most studied member, and TSP-2), and subgroup B (including TSP-3, TSP-4 and COMP); - TSP-1 is a multifunctional glycoprotein stored in the extracellular matrix; - TSP-1 is a homotrimer of three glycopeptides linked through disulfide bonds. The amino-terminal domain of TPS-1 has heparin-binding activity; the carboxyl terminal domain has cell-binding and calcium ion binding activity and between them there are domains with procollagen, properdin and EGF amino-acid homology.	- TPS-1 is recognized by at least 12 adhesion receptor (CD36, αv integrins, β1 integrins, etc.) and proteases involved in angiogenesis (plasminogen, urokinase, matrix metalloproteinase, etc.)	- TPS-1 induces ECs’ apoptosis;	[39,45,46,74,75,76,77]
2-ME	- estradiol metabolite with no estrogenic activity	- 2-ME binds poorly to estrogen receptors	- inhibits ECs’ proliferation and induces apoptosis in ECs.	[39,78,79]

Abbreviations: MMPs—Matrix metalloproteinases; EC—endothelial cell; ATP—adenosine triphosphate; TSPs—Thrombospondins; EGF—Epidermal growth factor; COMP—cartilage oligomeric matrix protein; 2-ME—2-Methoxyestradiol.

**Table 3:** Angiogenesis inhibitors approved to treat cancer in humans.

Angiogenesis Inhibitor Generic Name (Trade Name)	Description/Chemical Taxonomy	Mechanism of Action [102]	Approved to Treat (Alone or with Other Drugs) [101]
Axitinib (Inlyta^®^) https://go.drugbank.com/drugs/DB06626, accessed on 4 July 2022	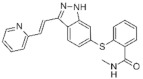	Axitinib selectively blocks the tyrosine kinase receptors VEGFR-1, VEGFR-2, and VEGFR-3.	Renal cell carcinoma [103]
Bevacizumab (Avastin^®^, Mvasi^®^, Zirabev^®^) https://go.drugbank.com/drugs/DB00112 accessed on 4 July 2022	Recombinant humanized monoclonal antibody	VEGF-A inhibitor	Cervical and colorectal cancer, glioblastoma, hepatocellular carcinoma, Non-squamous non-small cell lung cancer, Ovarian epithelial, fallopian tube or primary peritoneal cancer, Renal cell carcinoma [104]
Cabozantinib (Cometriq^®^) https://go.drugbank.com/drugs/DB08875 accessed on 4 July 2022	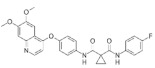	Non-specific receptor tyrosine kinase inhibitor	Hepatocellular carcinoma, Medullary thyroid cancer, Renal cell carcinoma [105]
Everolimus (Afinitor^®^) https://go.drugbank.com/drugs/DB01590 accessed on 4 July 2022	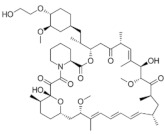	Everolimus works similarly to Rapamycin, being a derivative of Rapamycin (sirolimus). After binding to FKBP-12, Everolimus inhibits the activation of mTOR, a key regulatory kinase.	Breast, pancreatic, gastrointestinal and lung cancer, renal cell carcinoma, subependymal giant cell astrocytoma [106]
Lenalidomide (Revlimid^®^) https://go.drugbank.com/drugs/DB00480 accessed on 4 July 2022	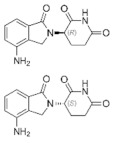	Being an analogue of thalidomide, Lenalidomide works through various mechanisms of action, promoting malignant cell death and enhancing host immunity.	Anemia, Follicular lymphoma, mantle cell lymphoma, marginal zone lymphoma, multiple myeloma [107]
Lenvatinib mesylate (Lenvima^®^) https://go.drugbank.com/drugs/DB09078 accessed on 4 July 2022	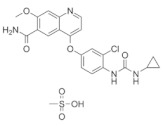	Receptor tyrosine kinase inhibitor	Endometrial carcinoma, hepatocellular carcinoma, renal cell carcinoma, thyroid cancer [108]
Pazopanib (Votrient^®^) https://go.drugbank.com/drugs/DB06589 accessed on 4 July 2022	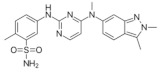	Second-generation multitargeted tyrosine kinase inhibitor	Renal cell carcinoma; Soft tissue sarcoma [109]
Ramucirumab (Cyramza^®^) https://go.drugbank.com/drugs/DB05578 accessed on 4 July 2022	Human monoclonal antibody (IgG1) against vascular endothelial growth factor receptor 2 (VEGFR2)	Ramucirumab is a direct VEGFR-2 antagonist, that blocks the binding of natural VEGF ligands.	Colorectal cancer, Hepatocellular carcinoma, Non-small cell lung cancer, Stomach adenocarcinoma or gastroesophageal junction adenocarcinoma [110]
Regorafenib (Stivarga^®^) https://go.drugbank.com/drugs/DB08896 accessed on 4 July 2022	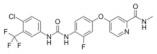	Multiple kinases inhibitor	Colo-rectal cancer, Gastrointestinal stromal tumor, Hepato-cellular carcinoma [111]
Sorafenib (Nexavar^®^) https://go.drugbank.com/drugs/DB00398 accessed on 4 July 2022	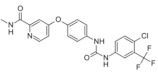	Kinase inhibitor	Hepatocellular carcinoma, Renal cell carcinoma, Thyroid cancer [112]
Sunitinib (Sutent^®^) https://go.drugbank.com/drugs/DB01268 accessed on 4 July 2022	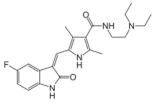	Receptor tyrosine kinase inhibitor	Gastrointestinal stromal tumor; Pancreatic cancer; Renal cell carcinoma [113]
Thalidomide (Synovir, Thalomid^®^) https://go.drugbank.com/drugs/DB01041 accessed on 4 July 2022	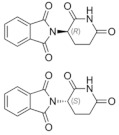	As a cancer treatment, thalidomide may act as a VEGF inhibitor.	Multiple myeloma [114]
Vandetanib (Caprelsa^®^) https://go.drugbank.com/drugs/DB05294 accessed on 4 July 2022	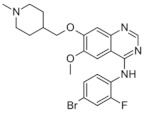	Tyrosine kinases inhibitor	Medullary thyroid cancer [115]
Ziv-aflibercept (Zaltrap^®^) https://go.drugbank.com/drugs/DB08885 accessed on 4 July 2022	Recombinant protein composed of the binding domains of two human VEGFRs fused with the Fc region of human IgG1.	VEGF inhibitor	Metastasized colorectal cancer [116]

Abbreviations: VEGF: vascular endothelial growth factor; VEGFR: Vascular endothelial growth factor receptor; FKBP-12: FK Binding Protein-12; mTOR: mammalian target of Rapamycin; IgG1: immunoglobulin gamma 1.

## Data Availability

All data included in this study are identified within the text, tables, and figures.
